# Integrative psychotherapy of patients with schizophrenic spectrum disorders

**DOI:** 10.1192/j.eurpsy.2021.1321

**Published:** 2021-08-13

**Authors:** R.M. Holm-Hadulla

**Affiliations:** Psycho-social Medicine, Heidelberg University, Heidelberg, Germany

**Keywords:** method integration, common and specific factors, schizophrenic spectrum disorders, psychotherapy

## Abstract

**Introduction:**

Psychotherapy of patients suffering from schizophrenic disorders remains controversial. There are promising descriptions of psychothereputic interventions in combination with pharmacological treatment. Some empirical studies show that different forms of psychotherapy are effective. However, there are few models to combine different psychotherapeutic strategies in a comprehensive way.

**Objectives:**

We here propose a model of integrative psychotherapy that is based on the therapeutic alliance and the general principles of understanding and communication. It comprises interpersonal, behavioral, psychodynamic, and existential elements.

**Methods:**

Theoretical principles of different psychotherapeutic schools are applied hermeneutically to a case of schizophrenia. Thus, general methods of psychotherapy gain contextual meaning by the analysis of an individual narrative. The patient himself assessed and approved his case report.

**Results:**

After remission of the acute symptoms, integrative psychotherapy played the major role in the recovery of a young musician. It is shown how the therapeutic alliance and communication in a general sense interacted with behavioral, cognitive, psychodynamic, and existential techniques. It is demonstrated narratively how recovery was facilitated, adjunct to pharmacological treatment, by method-integrative psychotherapy. Basic cognitive-behavioral techniques ensured at first the stabilization of the patient. Then psychodynamic remembering, repeating, and working through became possible. Finally, existential topics and creative solutions dominated the sessions.

**Conclusions:**

Psychotherapeutic method-integration is necessary to treat patients with various mental disorders especially schizophrenia. We propose an ABCDE-Model that comprises the following principles: Therapeutic alliance (A); behavior change (B); cognitive reflection on dysfunctional meanings and beliefs (C); psychodynamic remembering, repeating, and working through (D); and existential understanding and communication (E).

